# Effect of Air Storage on Stress Corrosion Cracking of ZK60 Alloy Induced by Preliminary Immersion in NaCl-Based Corrosion Solution

**DOI:** 10.3390/ma15217862

**Published:** 2022-11-07

**Authors:** Evgeniy Merson, Vitaliy Poluyanov, Pavel Myagkikh, Dmitri Merson, Alexei Vinogradov

**Affiliations:** 1 Institute of Advanced Technologies, Togliatti State University, 445020 Togliatti, Russia; 2 Department of Mechanical and Industrial Engineering, Norwegian University of Science and Technology, 4791 Trondheim, Norway

**Keywords:** air storage, corrosion products, fractography, Mg alloys, pre-exposure, stress corrosion cracking

## Abstract

The preliminary exposure of Mg alloys to corrosion solutions can cause their embrittlement. The phenomenon is referred to as pre-exposure stress corrosion cracking (PESCC). It has been reported that relatively long storage in air after pre-exposure to the corrosion solution is capable of eliminating PESCC. This effect was attributed to the egress of diffusible hydrogen that accumulated in the metal during pre-exposure. However, recent findings challenged this viewpoint and suggested that the corrosion solution retained within the side surface layer of corrosion products could be responsible for PESCC. The present study is aimed at the clarification of the role of hydrogen and the corrosion solution sealed within the corrosion products in the “healing” effect caused by post-exposure storage in air. Using the slow strain rate tensile (SSRT) testing in air and detailed fractographic analysis of the ZK60 specimens subjected to the liquid corrosion followed by storage in air, we found that PESCC was gradually reduced and finally suppressed with the increasing time and temperature of air storage. The complete elimination of PESCC accompanied by recovery of elongation to failure from 20% to 38% was achieved after 24 h of air storage at 150–200 °C. It is established that the characteristic PESCC zone on the fracture surface is composed of two regions, of which the first is always covered by the crust of corrosion products, whereas the second one is free of corrosion products and is characterised by quasi-brittle morphology. It is argued that the corrosion solution and hydrogen stored within the corrosion product layer are responsible for the formation of these two zones, respectively.

## 1. Introduction

Magnesium alloys being tensile tested in air after preliminary exposure to corrosive media suffer from a significant drop in mechanical properties, which is accompanied by a change in the fracture mode from ductile to brittle [1,2,3,4,5]. This phenomenon is known as pre-exposure stress corrosion cracking (PESCC) [6], also referred to as pre-exposure embrittlement [7,8]. Since the specific conditions inducing PESCC are less common in practice than those responsible for true stress corrosion cracking (SCC), significantly less attention has been paid to the former phenomenon. Nevertheless, understanding the mechanisms governing PESCC is of great importance because it sheds light on the nature of SCC, which is still under debate. For example, the widespread belief that hydrogen plays a key role in the mechanism of SCC [9,10,11,12] is primarily based on the observed similarity between the features of PESCC and hydrogen embrittlement (HE) in other metallic materials [13]. It was established that the degree of PESCC in many Mg alloys increases with decreasing strain rate [6,14] and increasing time of pre-exposure in various corrosion solutions [7,8,15,16,17,18,19,20]. Longer pre-exposure was found to cause more significant corrosion damage [18,19,20] and a larger brittle-like area on the peripheral part of the fracture surface [7,16,20], accompanied by an increase in the hydrogen concentration in the test specimens [14,20]. It was suggested [5,7,8,14,16,17,18] that hydrogen, being an inevitable by-product of a corrosion reaction, is absorbed by the metal in the form of atoms having a high diffusion rate that is sufficient to cause hydrogen-assisted cracking through the well-known HE mechanisms, including hydrogen-enhanced decohesion, hydrogen-enhanced localised plasticity, or delayed hydride cracking. The details of these mechanisms can be found elsewhere [21,22]. What should be stressed here is that all of them require the presence of a sufficiently high concentration of diffusible hydrogen in the metal matrix. A recent study has challenged this belief and shown that the role of diffusible hydrogen in PESCC can be substantially less important than that of the corrosion product layer deposited on the side surface of the metal during pre-exposure [6,20,23]. In particular, it was reported that PESCC of the alloys ZK60 and AZ31 preliminarily immersed in the NaCl-based corrosion solutions was completely eliminated by the chemical removal of the corrosion product layer, provided that irreversible corrosion damage was not too high [20,23]. Furthermore, a negligible concentration of diffusible hydrogen was found in the base metal of these alloys after the removal of corrosion products [20,23,24,25]. It was, therefore, suggested that the corrosion product layer may serve as a sealant for embrittling agents, such as hydrogen or retained corrosion solution, which facilitate the propagation of the brittle cracks under external loading [6,20,23]. The presence of the corrosion solution inside this layer is indicated by the crust of corrosion products, which was observed on the peripheral part of the fracture surface of the alloy ZK60 embrittled due to PESCC [6,16,20,23]. Originally, this crust was attributed to corrosion damage, which is induced during pre-exposure [16,23]. However, it was demonstrated recently that these corrosion products were formed not during the pre-exposure process but exactly during post-exposure mechanical testing in air [6]. Two independent experimental observations corroborated this finding. Firstly, the size of the area covered by corrosion products on the fracture surface decreased with an increasing strain rate. Secondly, many secondary cracks with corrosion products inside were found in the cross-section of the pre-exposed specimens after tensile testing, while none were observed right after pre-exposure. Obviously, this would not be the case if the cracks were present with the corresponding region of the fracture surface covered with the crust of corrosion products in the specimens before tensile testing. Thus, it was concluded that there must be an aggressive medium retaining inside the corrosion product layer on the side surface to form the corrosion products on the fracture surface. Later on, the presence of the corrosion solution within the corrosion product layer was additionally evidenced by the evolution of extra hydrogen generated by the corrosion reaction inside the specimens after pre-exposure to corrosive media [26].

The suggestion that the corrosion product layer containing the corrosion solution inside can be responsible for PESCC of Mg alloys prompted us to review this topic with new experimental findings relevant to PESCC, which has been exclusively associated with the effect of hydrogen so far. In particular, it was reported that vacuum annealing [14] or long exposure to dry air [5,7] after immersion in corrosion solution suppresses PESCC of Mg alloys to a certain extent. Chakrapani and Pugh [14] showed that vacuum annealing at 385 °C for 4 h results in a slight recovery of both the elongation to failure and ultimate tensile strength of the Mg-7.5%Al alloy embrittled by pre-exposure to the NaCl-K_2_CrO_4_ solution. The observed recovery of mechanical properties was attributed to the reduction in the hydrogen concentration in the specimens, as was assessed by the gas analyser after annealing. Moreover, the positive strain rate sensitivity of mechanical properties, which is a characteristic feature of HE in many alloys [27,28,29], was eliminated by annealing. This observation was considered as an additional argument blaming hydrogen for being the root cause of PESCC. Similar partial recovery of mechanical properties was documented after storing the pre-exposed specimens of pure Mg [5] and AZ31B [7] alloy in a desiccator. This was also explained by the removal of hydrogen from the specimens. Nevertheless, it was found recently that the complete elimination of PESCC in ZK60 can be achieved by the removal of the side surface corrosion product layer [6]. Concurrently, the strain rate sensitivity of ductility changed from positive to negative. Bearing in mind that, besides hydrogen, the corrosion solution can be contained within the corrosion product layer, one can plausibly suppose that the suppression of PESCC by air or vacuum exposure at room or elevated temperatures could be attributed not only to the extraction of hydrogen but also to the evaporation of water from the corrosion products. Thus, the primary aim of the present study was to clarify the significance and the role of post-exposure storage in air in the suppression of PESCC of the Mg alloy ZK60. To be more specific, we endeavour to establish relationships, or at least correlations, between mechanical properties, hydrogen concentration, and fractographic characteristics, including the area of fracture surface covered by corrosion products. To this end, the slow-strain rate tensile (SSRT) testing coupled with thermal desorption analysis of hydrogen and detailed quantitative fractographic and side surface examination were utilised. The ZK60 alloy was chosen as a typical representative of a class of wrought Mg alloys suffering from high susceptibility to PESCC. The grain microstructure and distribution of primary and secondary phases in the commercial hot-extruded alloy used in this work were documented in detail in the previous reports [24,25,30,31] by the present and other investigators; interested readers are encouraged to review the above-cited publications. 

## 2. Materials and Methods

The hot-extruded commercial alloy ZK60 was used in the present investigation in the as-received state. The chemical composition, microstructure, and the manufacturing process of the alloy were the same as reported in the previous studies [6,20,23,24]. The microstructure of the alloy is represented by α-Mg fine grains with a 3 µm average diameter and numerous secondary phase particles [24]. The cylindrical threaded specimens for SSRT testing, as well as the samples for the gas analysis, were machined from round bars of 25 mm diameter along the extrusion direction. The samples for the gas analysis and the gauge parts of the specimens for tensile testing had the same geometry and dimensions of 30 mm length and 6 mm diameter. All specimens and samples were annealed in vacuum at 250 °C for 2 h to relieve residual stresses. 

Before SSRT testing, the gauge part of the specimens was placed into the plexiglass cell, which was then filled with the 4% NaCl (NevaReactiv, St. Petersburg, Russia) + 4% K_2_Cr_2_O_7_ (ChimProm, Novocheboksarsk, Russia) corrosive solution. After 1.5 h of pre-exposure in this solution at an open-circuit potential without external stress, the specimens were extracted from the cell, cleaned with ethanol, and dried with compressed air. The pre-exposure was optionally followed by removal of corrosion products, which was executed by submerging the specimens in the standard 20% CrO_3_ (ChimProm, Novocheboksarsk, Russia) + 1% AgNO_3_ (NevaReactiv, St. Petersburg, Russia) aqueous solution for 1 min, followed by rinsing the specimens with ethanol (NevaReactiv, St. Petersburg, Russia) and drying by compressed air. Several pre-exposed specimens (with and without corrosion products) were SSRT tested within two minutes after pre-exposure, while others, including the reference ones, which did not undergo pre-exposure, were subjected to storage in ambient air for 24 h at temperatures ranging from 24 to 200 °C. The uniaxial SSRT testing of all specimens was performed in air at 24 °C with the 5 × 10^−6^ s^−1^ nominal strain rate (0.01 mm/min traverse velocity) using the AG-X Plus (Shimadzu, Kyoto, Japan) screw-driven frame. The samples for the gas analysis underwent the same treatments as their counterparts for the SSRT testing.

The fracture and side surfaces of the SSRT tested specimens were investigated by a scanning electron microscope (SEM) (SIGMA, Zeiss, Jena, Germany) equipped with an energy dispersive X-ray (EDS) detector (EDAX, Mahwah, NJ, USA). The quantitative geometrical measurements of the specific areas on the fracture surfaces were conducted either by the ImageJ (v1.52u, NIH, Bethesda, MD, USA) freeware or by the built-in SmartSEM (v05.04.05.00, Zeiss, Jena, Germany) software.

The thermal desorption of hydrogen from the pre-exposed samples was investigated by the G8 Galileo (Bruker, Kalkar, Germany) gas analyser via the hot extraction method in N_2_ (99.999%, LindeGas Rus, Samara, Russia) carrier gas flux. The concentration of hydrogen in the equipment was measured by the difference in the thermal conductivity of the pure carrier gas and the mixture of carrier gas with the extracted hydrogen. The contaminating admixtures such as CO_2_ and water were removed from the gas mixture by the system of filters. The pre-exposed samples were placed into the quartz tube of the gas analyser within 2 min after pre-exposure or after 24 h of air storage at a specific temperature. The procedure of the thermal desorption analysis (TDA) included (i) heating the sample from room temperature of 24 °C to 450 °C with a constant heating rate of 38 °C/min, followed by (ii) 15 min of baking at 450 °C and (iii) cooling for 10 min. The additional details of the gas analysis procedure have been thoroughly described elsewhere [24].

## 3. Results and Discussion

### 3.1. Mechanical Properties

**Figure 1:**
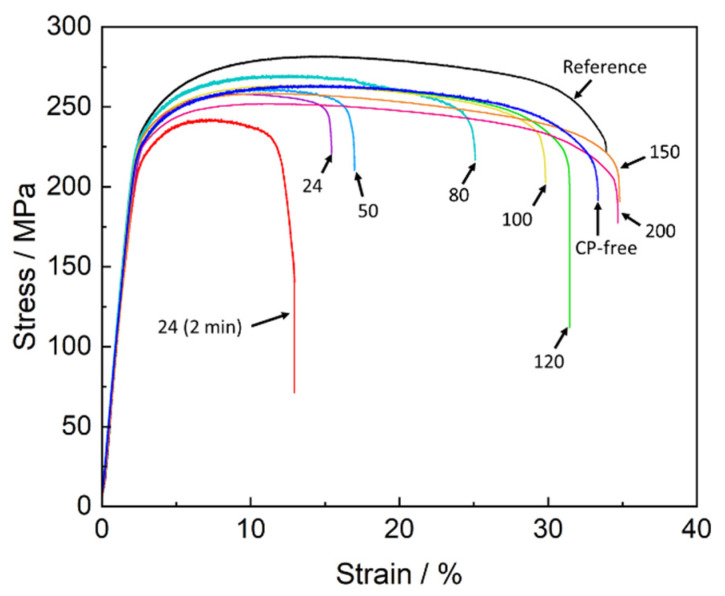
Stress–strain diagrams corresponding to the specimens of the ZK60 alloy which were SSRT tested in the reference and CP-free state as well as after pre-exposure to the corrosion solution followed by storage in air. Temperatures of air storage (in °C) are provided at the arrows. The duration of storage in air was 24 h, if not marked otherwise.

**Figure 2:**
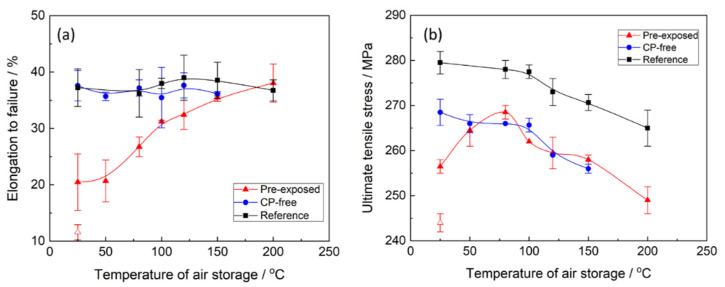
The effect of temperature of air storage on elongation to failure (**a**) and ultimate tensile strength (**b**) of the reference, CP-free, and pre-exposed specimens of the ZK60 alloy. The open and filled symbols indicate the pre-exposed specimens after air storage for 2 min and 24 h, respectively.

The mechanical testing showed that the specimens which were SSRT tested in air within a few minutes after pre-exposure to the corrosion solution demonstrated a substantial loss in strength and ductility compared to the reference specimens (Figure 1 and Figure 2). This is typical behaviour for ZK60 embrittled by PESCC. In harmony with our previous reports [6,20,23], the removal of corrosion products resulted in complete recovery of ductility and partial recovery of strength in all specimens tested. The apparent irreversible loss of strength, which remains after the removal of corrosion products, is not related to embrittlement but rather is due to the reduction in the specimens’ cross-section caused by irreversible corrosion damage [20]. It was found that similarly to the removal of corrosion products, post-exposure air storage inhibits PESCC. The efficiency of this recovery process, however, depends on the time and temperature of the storage of specimens in air. As can be seen in Figure 1 and Figure 2, both ultimate tensile strength (UTS) and elongation to failure (EF) of the pre-exposed specimens after air storage at 24 °C for 24 h are significantly higher than those after 2 min of air storage at the same temperature. Nevertheless, even after 24 h of storage at room temperature, the pre-exposed specimens show roughly 50% loss of EF and approximately 25 MPa smaller UTS in comparison with their reference counterparts. It was found that the recovery of mechanical properties can be substantially enhanced by storage of the specimens in air at elevated temperatures. Figure 2a demonstrates that the increased temperature of air storage results in the increase in EF. The improvement in the EF value is non-monotonic and is most pronounced in the temperature range from 50 to 100 °C. After storage in air at 100 °C, the ductility loss of the pre-exposed specimens does not exceed 20%. However, at a chosen post-exposure storage time, the complete recovery of ductility can be achieved only at 150–200 °C. After such treatment, the measured EF values were the same as those of the reference specimens and the specimens with the removed corrosion product layer (referred to hereafter as CP-free specimens).

The effect of storage temperature on the strength of the pre-exposed specimens is more complex than on ductility. It can be seen from Figure 2b that UTS of the specimens monotonically grows when the storage temperature increases from 25 to 80 °C, while the further temperature increase up to 200 °C results in the non-monotonic decrease in UTS with a short plateau around 120–150 °C. To clarify whether the observed complex behaviour of strength and ductility is associated with temperature-induced microstructural changes or if it is attributed specifically to PESCC, the effect of annealing temperature on mechanical properties of the reference and CP-free specimens was investigated. One can see (Figure 2b) that the UTS of these specimens is almost unaffected by low-temperature annealing below 100 °C, while at higher temperatures, the strength decreases monotonically with temperature. Apparently, some ageing-related microstructural changes affecting the strength occur in the alloy at temperatures above 100 °C. Therefore, these changes are likely to take some part in the observed deterioration of UTS in the pre-exposed specimens subjected to air storage above 100 °C. However, the opposite trend, i.e., the increase in UTS, in the pre-exposed specimens stored in air in the temperature range below 80 °C, cannot be explained by any microstructural changes in the metallic matrix. Furthermore, the decrease in strength in the pre-exposed specimens, which is observed at higher temperatures, cannot be solely attributed to the microstructural transformations either, because it starts in the range between 80 and 100 °C and not between 100 and 120 °C as for the reference and CP-free specimens. Furthermore, the plateau between 120 and 150 °C featuring the dependence of UTS on the temperature of the pre-exposed specimens is absent on such dependencies for the specimens of other kinds. Thus, the other factors responsible for the decrease in UTS, which is attributed specifically to the specimens embrittled by pre-exposure, do exist. It is important to note that the microstructural changes, which likely affect the strength of the alloy at elevated temperatures, do not influence the ductility. It follows from Figure 2a that the EF of the reference and CP-free specimens scatters randomly around the nearly constant average value within the whole investigated temperature range. Therefore, the increasing EF of the pre-exposed specimens with post-exposure storage temperature should be entirely associated with the elimination of PESCC.

### 3.2. Fractographic and Side Surface Observations

**Figure 3:**
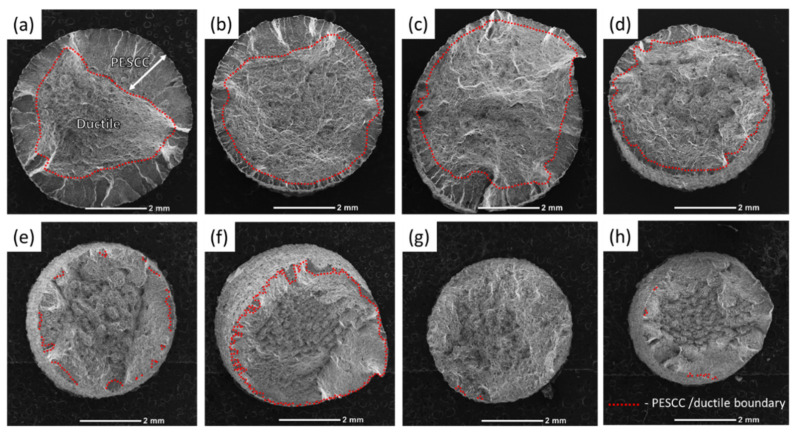
Whole views of the fracture surfaces of the specimens SSRT tested after pre-exposure followed by storage in air at 24 °C for 2 min (**a**) and by 24 h, at 24 °C (**b**), 50 °C (**c**), 80 °C (**d**), 100 °C (**e**), 120 °C (**f**), 150 °C (**g**), and 200 °C (**h**).

**Figure 4:**
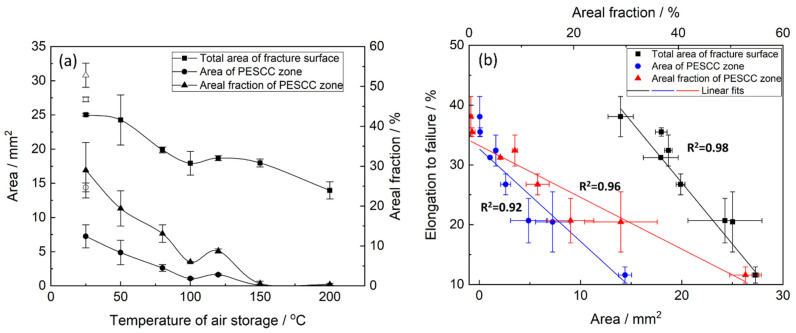
The effect of temperature of post-exposure storage on the areal fractographic characteristics of the ZK60 specimens tested after pre-exposure followed by air storage (**a**) and the correlation between these characteristics and the elongation to failure of the specimens (**b**). The open and filled symbols correspond to the pre-exposed samples subjected to air storage for 2 min and 24 h, respectively.

The fracture surface of the specimens suffering from PESCC is characterised by the peripheral brittle-like “PESCC zone” surrounding the dimpled ductile region in the central part of the fracture surface. The similar appearance of the fracture surface was frequently reported to be characteristic of the pre-exposure effects in Mg alloys, including ZK60, used in the present study [7,16,23]. The quantitative fractographic analysis showed that, in general, the increase in the time and temperature of post-exposure storage in air results in the significant reduction in both the total area of the fracture surface and the area of the PESCC zone (as well as its areal fraction with respect to the total area of the fracture surface) (c.f., Figure 3 and Figure 4a). It also follows from Figure 3f–h that when the temperature of air storage exceeds 80 °C, the PESCC zone becomes discontinuous and is represented by several small isolated “islands” with a brittle-like morphology at the edge of the fracture surface. After post-exposure storage at 150–200 °C, the PESCC zone is almost completely absent. However, some specimens occasionally exhibited a few tiny areas with the brittle topology (e.g., Figure 3g,h) even after storage at 150–200 °C. Despite the generally increasing apparent ductility of the fracture surface with the temperature of post-exposure storage, one can notice the anomalous increase in the fraction of the brittle relief on the fracture surface after storage at 120 °C (Figure 4a). As was shown above, the decrease in strength was supposedly associated with the microstructural changes occurring at the same temperature in the reference specimens.

The fractographic observations corroborate well with the results of mechanical testing showing the increasing ductility of the pre-exposed specimens due to the suppression of PESCC by post-exposure storage in air. The confident linear correlation with Pearson’s R^2^ > 0.91 is found between EF and the total area of their fracture surface as well as the area of the PESCC zone and its areal fraction (Figure 4b), thus confirming the link between the propagation of PESCC and the material’s embrittlement unveiled by SSRT testing in ambient conditions.

**Figure 5:**
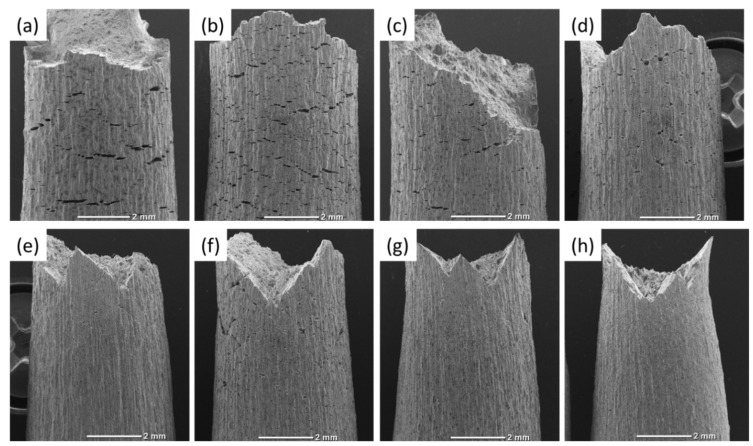
SEM images of side surfaces of the specimens SSRT tested after pre-exposure followed by air storage at 24 °C for 2 min (**a**), and by 24 h at 24 °C (**b**), 50 °C (**c**), 80 °C (**d**), 100 °C (**e**), 120 °C (**f**), 150 °C (**g**), and 200 °C (**h**).

**Figure 6:**
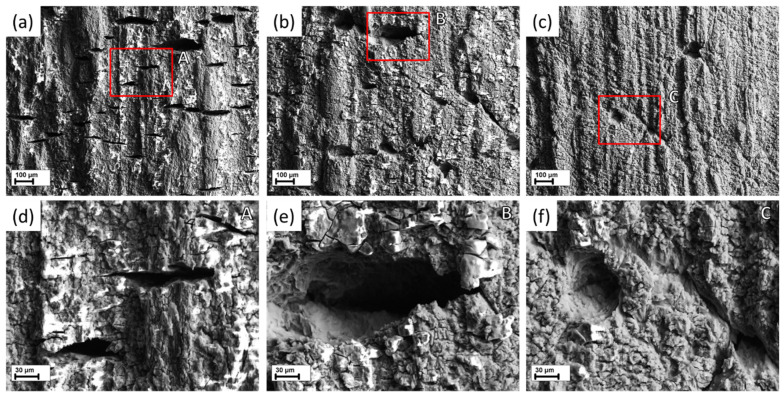
The appearance of the side surface cracks in the specimens SSRT tested after pre-exposure followed by 24 h of air storage at: 24 °C (**a**,**d**), 80 °C (**b**,**e**), and 100 °C (**c**,**f**). The magnified SEM images of the regions outlined by the frames A–C in (**a**–**c**) are represented in (**d**–**f**) to show the microscopic characteristics of side surface cracks.

**Figure 7:**
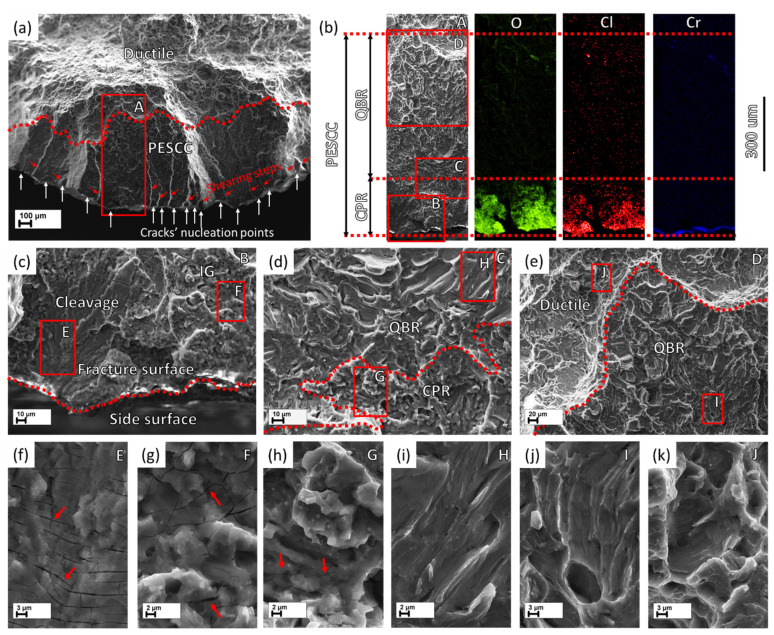
The fractographic features of the specimen SSRT tested after pre-exposure followed by 24 h of air storage at 24 °C: (**a**) the peripheral region of the fracture surface with PESCC zone delimited from the ductile region by the dotted curve. The vertical white and inclined red arrows indicate the nucleation points of the multiple cracks and the shearing ligaments between them, respectively; (**b**) the magnified SEM image with elemental EDS maps of the region outlined by the frame A in (**a**) showing the boundaries of the specific regions; (**c**) the magnified SEM image of the region outlined by the frame B in (**b**) showing the crack’s nucleation part of the corrosion product region (CPR) characterised by cleavage and intergranular facets and corrosion products; (**d**) the magnified SEM image of the region outlined by the frame C in (**b**) showing the transition region between CPR and the quasi-brittle region (QBR); (**e**) the magnified SEM image of the region outlined by the frame D in (**b**) showing the transition region between QBR and the ductile region; (**f**–**k**) the magnified SEM images of the regions outlined by the frames E–J in (**c**–**e**) showing the characteristic morphologies of the specific regions.

**Figure 8:**
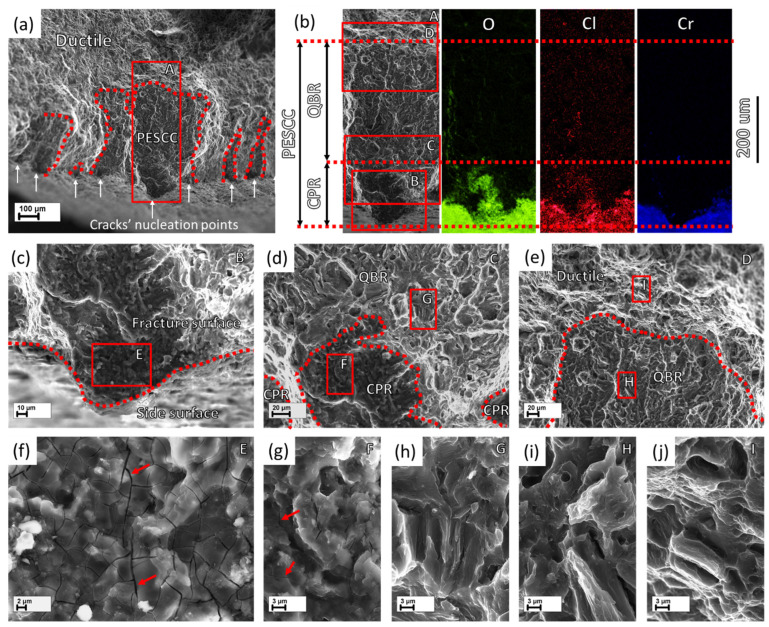
The fractographic features of the specimen SSRT tested after pre-exposure followed by 24 h of air storage at 80 °C: (**a**) the peripheral region of the fracture surface with isolated islands of PESCC zone delimited from the ductile region by the dotted curve. The vertical white arrows indicate the nucleation points of the multiple cracks; (**b**) the magnified SEM image with elemental EDX maps of the region outlined by the frame A in (**a**) showing the boundaries of the specific regions; (**c**) the magnified SEM image of the region outlined by the frame B in (**b**) showing the crack’s nucleation part of CPR; (**d**) the magnified SEM image of the region outlined by the frame C in (**b**) showing the transition region between CPR and QBR; (**e**) the magnified SEM image of the region outlined by the frame D in (**b**) showing the transition region between QBR and ductile region; (**f**–**j**) the magnified SEM images of the regions outlined by the frames E–I in (**c**–**e**) showing the morphologies characteristic of the specific regions.

**Figure 9:**
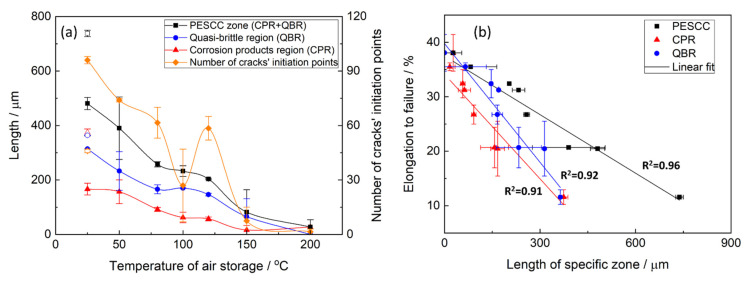
The effect of temperature of post-exposure storage on the linear fractographic characteristics of the ZK60 specimens tested after pre-exposure followed by air storage (**a**) and the correlation between these characteristics and the elongation to failure of the specimens (**b**). The open and filled symbols correspond to the pre-exposed samples subjected to air storage for 2 min and 24 h, respectively. CPR—the region covered by the crust of corrosion products, QBR—the quasi-brittle region free of corrosion products.

The side surface observations in combination with the fractographic analysis show that the PESCC zone is formed due to the propagation of multiple cracks nucleating at the side surface along the whole gauge part of the specimens (c.f., Figure 5 and Figure 6). These cracks coalesce during their propagation towards the specimen’s centre as well as in the transverse direction. The coalescence of the cracks occurs in a stepwise manner by mutual axial shearing of the two halves of the fracture surface. The location of the cracks’ nucleation sites on the peripheral part of the fracture surface corresponds to relatively flat regions separated from each other by the axial shearing steps. As an example, the shearing steps and the crack initiation sites between them are indicated by the inclined red and vertical white arrows, respectively, in Figure 7a and Figure 8a. As is seen in the SEM side surface images in Figure 5 and Figure 6, the number and size of the side surface cracks decrease considerably with the temperature of post-exposure storage. This visual observation is testified by the results of the quantitative fractographic analysis provided in Figure 9a. According to these results, the number of cracks’ nucleation sites on the fracture surface increases considerably along with the storage temperature. Furthermore, as the temperature increases, the side surface cracks become shorter in the transverse direction and acquire a more ductile appearance, as is indicated by their less sharp and more round geometry (c.f., Figure 6). It follows from Figure 5a,b and Figure 9a that the increase in the time of post-exposure storage in air at 24 °C from 2 min to 24 h results in the remarkable growth in the number of side surface cracks and the cracks’ nucleation sites on the fracture surface. Thus, the number of cracks decreases with temperature and increases with the time of air storage. It is worth noting that, similarly to the area of the PESCC zone, the number of the cracks’ nucleation sites exhibits a sharp increase after air storage at 120 °C (Figure 9a), whereas just a few cracks can be found in the specimens subjected to holding in air at 150–200 °C.

The PESCC zone in all pre-exposed specimens is composed of two distinct regions referred to as the corrosion product region (CPR) and the quasi-brittle region (QBR), which are seen one after the other from the edge of the specimen to the centre (Figure 7 and Figure 8). The corrosion product region starts to develop immediately at the edge of the fracture surface and is covered by the cracked crust of corrosion products (Figure 7b,c,f–h and Figure 8b,c,f,g). It has been shown recently that the intergranular cracking assisted by the corrosion solution sealed in the side surface layer of corrosion products can be responsible for the formation of the corrosion product region on the fracture surface [6]. Thus, the specific “melted” relief, which is observed within this region under the crust of corrosion products (Figure 7g,h and Figure 8f,g), is probably composed of fine intergranular facets attacked by corrosion. In addition, the large transgranular cleavage facets exhibiting corrosion damage can also be occasionally distinguished within the corrosion product region on the fracture surface (Figure 7c,f). The thickness of the corrosion product layer on the fracture surface decreases with the distance from the edge of the fracture surface towards the following quasi-brittle region, as is firmly evidenced by the EDX analysis (Figure 7b and Figure 8b). Additionally, the EDX elemental map obtained for the PESCC zone undeniably shows that this layer is strongly enriched with oxygen and chlorine. It should be stressed that the concentration of these elements decreases progressively along the crack path within the corrosion product region; however, it drops down abruptly to the negligible level at the boundary between the corrosion products and quasi-brittle regions. Thereby, the latter region is always completely free of traces of aggressive elements. Alternatively, the CPR–QBR boundary can be reliably distinguished by the morphological features in the SEM images with no aid from the EDX. The characteristic microcracks, such as those indicated by the arrows in Figure 7f–h and Figure 8f,g, are found to be indispensable attributes of the crust of corrosion products. As such, they serve as independent markers of corrosion products on the fracture surface. The size and density of these microcracks are gradually reduced with the thickness of the corrosion product layer throughout the respective region (see Figure 7f–h and Figure 8f,g). However, the cracks cease to appear in the quasi-brittle region beyond the CPR–QBR boundary (c.f., Figure 7i–k and Figure 8h–j). Moreover, in contrast to the region that has been covered by corrosion products, the morphology of the quasi-brittle region is distinctively characterised by various tear ridges, small dimples, and fluted facets (Figure 7j,i and Figure 8h,i). These features indicate the appreciable contribution of plastic deformation to the crack growth mechanism. 

**Figure 10:**
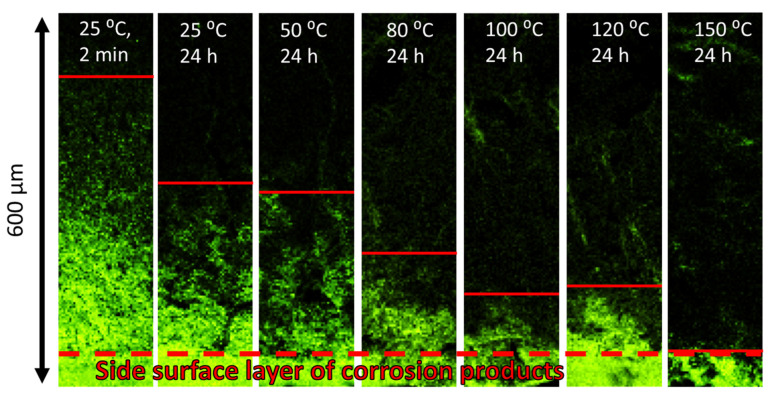
The distribution of oxygen on the peripheral part of the fracture surface for the specimens SSRT tested after pre-exposure followed by storage in air at different temperatures and durations. The red solid horizontal lines show the boundary between the CPR and QBR. The dashed line indicates the boundary between the side and fracture surfaces.

Notably, the CPR–QBR boundary recognised by the topological features on SEM images matches precisely with that distinguished by the EDX analysis (Figure 7b and Figure 8b). Thus, both methods can be used to characterise and identify two morphologically distinct regions. Apparently, the secondary electron imaging is less laborious in comparison with the EDX mapping. That is why the former was prioritised for further measurements. The length of the corrosion product zone, l_CPR_, as well as the length of the quasi-brittle region, l_QBR_, and the PESCC zone, l_PESCC_, were measured for each crack within the PESCC zone, as is schematically shown in Figure 7b and Figure 8b. The average values of these characteristics were calculated for the specimens tested under specific experimental conditions. The effect of air storage temperature and time on the measured fractographic properties is illustrated in Figure 9a: the lengths of all specific zones, including the corrosion product region, decrease significantly with temperature and time. In particular, the corresponding reduction in l_CPR_ is also evidently illustrated by the EDX oxygen maps shown in Figure 10. The obtained results corroborate the conclusions made in the previous study that the corrosion products on the fracture surface are produced during the SSRT testing. Indeed, if the corrosion product region was formed during pre-exposure, the size of this region would not be affected by subsequent air storage. It is worth noting that reduction in l_CPR_ becomes notable at the temperatures of air storage higher than 50 °C, while the l_QBR_ considerably decreases after storage at a lower temperature. In the temperature range of 100–120 °C, the plateau followed by the further decay up to near complete vanishing at higher temperatures of air storage is seen on the l_CPR_, l_QBR_, and l_PESCC_ curves in Figure 9a. It is established that the lengths of all three characteristic regions, including the corrosion product region, correlate well with the elongation to failure of the specimens embrittled by pre-exposure to the aggressive environment (Figure 9b). This finding suggests the crucially active role of the corrosion solution sealed/stored within the side surface layer of corrosion products in the mechanism of PESCC.

**Figure 11:**
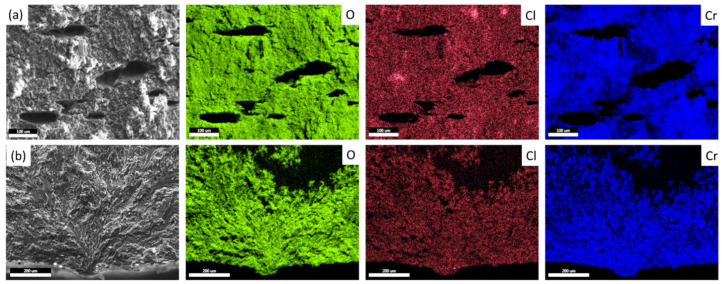
The SEM images and elemental maps obtained by EDX from the side surface of the specimen SSRT tested after pre-exposure in the 4% NaCl + 4% K_2_Cr_2_O_7_ corrosion solution (**a**) and from the fracture surface of the specimen SSRT tested in the same corrosion solution (**b**).

As has been mentioned, the corrosion products on the fracture surface can be associated with the corrosion solution, which is likely sealed within the corrosion product layer on the side surface. In conjunction with this suggestion, it is important to note that according to the results of EDX analysis, the corrosion product region on the fracture surface almost does not contain chromium (Figure 7b and Figure 8b), which is, however, abundantly present in the side surface layer of corrosion products (Figure 11a). This feature of the corrosion product region also drastically differs the fracture surface of the tensile tested pre-exposed specimens from that of the specimens which have been SSRT tested right in the corrosive solution containing chromates. The EDX maps obtained from the fracture surface of the specimen SSRT tested in the 4% NaCl + 4% K_2_Cr_2_O_7_ solution in the previous study [19] are provided in Figure 11b. It is clear that the fracture surface of this specimen demonstrates high concentrations of Cl, O, and Cr. This finding implies that the corrosion solution responsible for the formation of corrosion products on the fracture surface of the pre-exposed specimens is Cr-depleted and thus, its chemical composition can be different from that of the liquid initially used in the pre-exposure process.

### 3.3. Hydrogen Desorption Analysis

**Figure 12:**
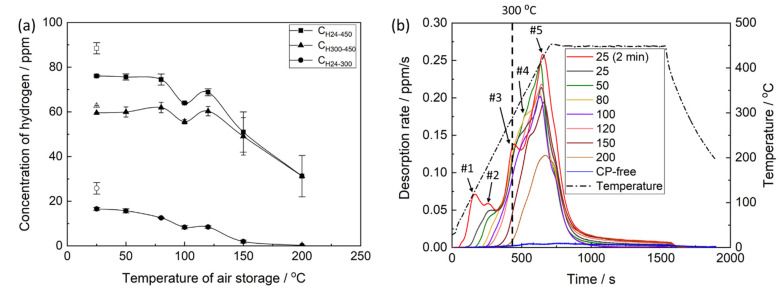
The effect of temperature and time of air storage on the concentration of hydrogen extracted in different temperature intervals (**a**) and on the thermal desorption spectra of hydrogen (**b**) for ZK60 subjected to pre-exposure followed by air storage at different temperatures. The open and filled symbols in (**a**) correspond to the pre-exposed samples subjected to air storage for 2 min and 24 h, respectively.

**Figure 13:**
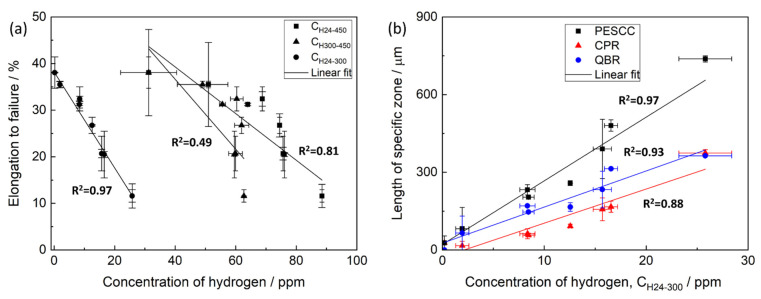
The correlation between the concentration of hydrogen extracted in different temperature intervals and the elongation to failure (**a**) and the lengths of specific zones on the fracture surface (**b**) of the specimens SSRT tested after pre-exposure followed by air storage at different temperatures and durations.

The thermal desorption analysis showed that the concentration of hydrogen extracted from the pre-exposed samples in the temperature range between 24 and 450 °C, *C_H24–450_*, decreases notably with increasing temperature and time of air storage. The dependence of *C_H24–450_* on the temperature of air storage is non-linear and is characterised by the plateau within the interval between 24 and 80 °C, followed by the remarkable decay at higher temperatures (Figure 12a). Moreover, the anomalous increase in the hydrogen concentration is observed after air storage at 120 °C. The correlation between *C_H24–450_* and elongation to failure of the pre-exposed samples subjected to air storage at different temperatures is poor (Figure 13a), indicating that the relationship between *C_H24–450_* and the degree of PESCC is unlikely. It is generally accepted that diffusible hydrogen, i.e., chemically free hydrogen possessing high mobility within a metal, is responsible for HE in steels and other alloys [32]. It has been well established that this hydrogen completely escapes from metals at temperatures below 300 °C [33]. As was shown in our previous reports, the concentration of diffusible hydrogen in the matrix of the pre-exposed specimens of ZK60 and AZ31 alloys was negligible [20,23]. However, chemically free hydrogen can be, probably, accumulated within the layer of corrosion products. The extraction temperature of this hydrogen is also expected to be below 300 °C. The hydrogen extracted from the metals at temperatures above 300 °C is usually considered to be immobile, and hence, its role in hydrogen-assisted cracking is relatively insignificant. Thus, the concentrations of hydrogen extracted from the pre-exposed samples in the temperature intervals of 24–300, *C_H24–300_,* and 300–450 °C, *C_H300–450_,* were additionally assessed using the thermal desorption curves shown in Figure 12b. It was found that the behaviour of *C_H300–450_*, depending on the temperature of air storage, is similar to that of *C_H24–450_* exhibiting the plateau at 24–80 °C, followed by the overall decay with the hump at 120 °C. In contrast, *C_H24–300_* notably decreases in the whole temperature range of air storage, including the 24–80 °C interval, where *C_H24–450_* and *C_H300–450_* remain almost unchanged. Furthermore, the concentration of hydrogen extracted below 300 °C during the thermal desorption analysis is negligible in the samples subjected to air storage at 150 and 200 °C. A strong correlation is found between *C_H24–300_* and EF of the pre-exposed specimens, which were exposed to air at different temperatures and times (Figure 13a), whereas the correlation between *C_H300–450_* and EF is even worse than that of *C_H24–450_*. Moreover, *C_H24–300_* appreciably correlates with the size of specific zones on the fracture surface of the pre-exposed specimens, including the PESCC zone, corrosion product region and quasi-brittle region (Figure 13b).

The thermal desorption spectra of hydrogen obtained from the specimens, which were subjected to pre-exposure, exhibit a few distinct superimposed peaks, as can be seen in Figure 12b. It was conclusively established in the previous studies [20,23,24] that all the observed peaks are associated with the corrosion products on the side surface layer because no considerable desorption of hydrogen from the matrix of the pre-exposed specimens occurs after chemical removal of those corrosion products. In support of this observation, the desorption spectrum for the specimen with the removed corrosion product layer is provided in Figure 12b (the plot is denoted as “CP-free”) for reference. Thus, all peaks observed on the thermal desorption diagram in Figure 12b should be associated either with chemically bonded hydrogen evolving from the thermally decomposing components of corrosion products or with the chemically free hydrogen. The latter can be either trapped in the corrosion product layer or produced during the corrosion of the Mg matrix interacting with the corrosion solution sealed within that layer. All hydrogen other than that linked to chemical components of corrosion products can be considered to be potentially capable for facilitating crack growth and, thus, inducing embrittlement associated with PESCC. The large peaks #3–5 appearing above 300 °C likely correspond to such components of corrosion products as Mg(OH)_2_ and/or MgH_2_, which decompose in the temperature range of 280–450 °C [13,34,35]. It can be seen that at least peaks #4 and #5 are still present on the desorption spectra after air storage at 150 and 200 °C, though PESCC is fully eliminated at these temperatures. Thus, hydrogen related to the high-temperature peaks #4 and #5 is not likely responsible for PESCC. This conclusion corroborates the fact that the concentration of hydrogen extracted in the temperature intervals 25–450 and 300–450 °C poorly correlates with the propagation of PESCC (Figure 13a). The low-temperature peak indicated as #1 in Figure 12b is attributed to hydrogen, which freely evolves from the CP layer at room temperature. This is evidenced by the absence of this peak on the thermal desorption spectra corresponding to the pre-exposed specimens subjected to air storage at 24 °C for 24 h. Moreover, the bubbles of hydrogen gas emanating from the pre-exposed specimen can be seen by the naked eye when this specimen is submerged in an inert liquid such as CCl_4_ [26]. This hydrogen can be associated with the molecular or atomic hydrogen liberated from the corrosion product layer as well as with hydrogen, which is produced in situ by the corrosion reaction [26]. Since the pre-exposed specimens do suffer from appreciable embrittlement after 24 h of air storage at 24 °C but do not exhibit peak #1 in the thermal desorption spectra, the hydrogen associated with this peak is not sufficient to be the sole reason for PESCC. Nevertheless, the contribution of this hydrogen to the observed embrittlement is possible because the reduction in peak #1 due to air storage at 24 °C is accompanied by the partial recovery of ductility. The nature of peaks #2 and #3 is still unknown. Presumably, they can be associated with the desorption of atomic or molecular hydrogen stored within the corrosion product layer or with the thermal decomposition of some hydrogen-containing components in this layer. As follows from Figure 12b, both peaks contribute considerably to the concentration of hydrogen extracted below 300 °C, which is found to be correlated with the extension of PESCC (Figure 13a), as well as with the size of the specific zones on the fracture surface (Figure 13b). Thus, hydrogen associated with peaks #1–3 can likely be involved in the PESCC phenomenon. However, the detailed analysis of origin and activation energies of hydrogen in different traps in the surface layer with corrosion products has yet to be carried out, e.g., methodologically similarly to that used in [36] to clarify the hydrogen distribution in the environmentally embrittled Al-Cu-Mg alloy.

### 3.4. Diffusible Hydrogen in the Matrix

The results of the present study show that PESCC of the alloy ZK60 can be completely eliminated by air storage of the specimens pre-exposed to the liquid corrosive environment. For the explanation of such a prominent effect of air storage, the contribution of a few factors, which can potentially affect the PESCC behaviour, is to be considered here.

Several authors suggested that the partial elimination of PESCC of Mg and its alloys caused by air storage in a desiccator at room temperature or by vacuum annealing at elevated temperatures can be attributed to the removal of diffusible hydrogen, which had been accumulated in the bulk metal during pre-exposure [5,7,10]. However, the concentration of diffusible hydrogen in the matrix of the pre-exposed specimens is negligible, as has been shown in the present and previous studies [16,19,20]. Additionally, let us reiterate that the ductility loss caused by PESCC can be completely eliminated by the removal of corrosion products from the side surface. Consequently, the absorption of diffusible hydrogen in the bulk of Mg being exposed to the corrosion solution is likely quite limited. In particular, it was suggested that the deep penetration of hydrogen into the metal is hindered by the surface layers of Mg hydroxide or hydride forming on the Mg surface during corrosion in aqueous solutions [20]. The diffusion rates of hydrogen in both these compounds are extremely low [9]. Thus, the PESCC phenomenon and its suppression by the storage in air can hardly be associated with the concentration of diffusible hydrogen in the matrix. 

### 3.5. The Corrosion Solution in the Corrosion Product Layer

The present and other reports [6,16,20,23] have documented that the peripheral part of the fracture surface of ZK60 specimens, which were SSRT tested in air after pre-exposure, is abundantly covered by corrosion products. Several independent experimental observations convincingly demonstrated that these products are created during the SSRT testing in air, when none of the external aggressive environments interact with the surface of the specimens. To be more specific, it is strongly supported by the following observations: (i) no secondary cracks containing corrosion products inside are found in the pre-exposed specimens before mechanical testing [6], and (ii) the length and area of corrosion product region on the fracture surface are reduced with the increasing strain rate [6] and (iii) the temperature of air storage [present study]. If the corrosion product regions were formed during pre-exposure, they would be observed in the cross-section of the specimen right after pre-exposure, while their size would not be affected by the strain rate or conditions of air storage. Assuming that ambient air, in which the SSRT testing is carried out, cannot cause the formation of extensive corrosion products, we can conclude that there must be some corrosive medium present in the side surface layer of corrosion products or at the interface between this layer and the surface of the specimen during SSRT testing. The interaction of this corrosive medium with the crack tip is, therefore, decided to be responsible for the formation of the corrosion product region on the fracture surface. Moreover, the correlation between the length of this region and the degree of PESCC observed in the present study suggests that the corrosion solution contained within the corrosion product layer plays a crucial role in the mechanism of PESCC. 

Although the nature of the corrosive medium being stored within the corrosion product layer is questionable and additional comprehensive investigation addressing this issue is required, some plausible explanations can be proposed. For example, it might be supposed that during pre-exposure, some amount of the original corrosion solution is sealed within the discontinuities, such as microcracks and voids, which are abundantly present within the corrosion product layer. Alternatively, the corrosive medium can probably be produced inside this layer after extraction of the specimen from the corrosion solution. It has been reported that the Mg hydroxide can react with CO_2_ from ambient air, leading to the formation of magnesite MgCO_3_ and water [37,38]. Probably, some components of corrosion products, such as MgCl_2_, can be dissolved in this water, thus making the solution even more aggressive. In favour of this scenario, one may recall the fact that the corrosion product region is always Cr-free. The original corrosion solution contains K_2_Cr_2_O_7_ producing the passive film on the Mg surface. The presence of this film is evidenced by the high concentration of Cr in the side surface layer of corrosion products in the pre-exposed specimens as well as on the fracture surface of the specimens tested in the corrosion solution. In contrast to MgCl_2_, the chromates, which are formed on the Mg surface, are sparingly soluble in water. Thus, the lack of Cr in the newly formed corrosion solution is explainable. The reaction of the saline water with the crack surface would produce MgO, Mg(OH)_2_, and MgCl_2_, providing the high concentration of oxygen and chlorine on the elemental maps obtained by EDX from the corrosion product region on the fracture surface. 

Regardless of its origin, the corrosion solution within the corrosion product layer should contain water to activate and maintain the electrochemical corrosion reaction producing corrosion products on the fracture surface. It can be suggested that air storage after pre-exposure is accompanied by the evaporation of this water from the corrosion product layer. The amount of evaporated water should grow with increasing temperature and time of air storage, causing consumption of the available corrosion solution. This can explain the reduction in corrosion product region on the fracture surface with time and temperature of air storage. As has been discussed above, the size of this region decreases progressively within the temperature range from 50 to 120 °C and almost vanishes after storage in air at 150–200 °C. The complete evaporation of water in this temperature range is well expected. The size of the corrosion product region is also reduced with the increasing time of air storage. However, unlike the effect of temperature, the longer time of air storage results in an increasing number of surface cracks. Probably, during longer post-exposure storage, more local volumes of hydroxide transform into magnesite with the release of water, thus creating a greater number of favourable sites for crack initiation. Nevertheless, the size of the corrosion product region decreases with the time of air storage because the total amount of the corrosion solution available at the cracks’ initiation sites decreases due to the evaporation of water.

Despite the obvious interaction of the corrosive medium with the surface of the propagating cracks during mechanical testing of the pre-exposed specimens, the exact role of this interaction in the mechanism of the crack growth remains unclear. It was shown previously that the corrosion product region is mainly produced by intergranular cracking [6]. For example, the anodic dissolution along the grain boundaries, which are enriched with noble secondary phase particles, might be responsible for this kind of fracture mode. On the other hand, the anodic dissolution of Mg is always accompanied by the cathodic reaction of hydrogen evolution. The adsorption of this hydrogen at the crack tip or its absorption within a few atomic layers beneath the surface can cause intergranular cracking, as well as transgranular cleavage through the HE mechanism, referred to as adsorption-induced dislocation emission (AIDE) [21,39]. Furthermore, the corrosion solution being in contact with the juvenile metal surface at the crack tip can possibly act as the surface active liquid, inducing the embrittlement through Rehbinder’s effect [40]. Thus, it is not necessary that the anodic dissolution accompanied by the formation of corrosion products on the fracture surface is the rate-controlling factor for the crack propagation.

### 3.6. Hydrogen in the Corrosion Product Layer

It is found that the corrosion product region is always followed by the quasi-brittle region, which exhibits no signs of any interaction with the corrosion solution. All features indicating the signs of corrosion on the fracture surface, including the elevated concentration of oxygen and chlorine as well as the microcracks attributed to the crust of corrosion products, cease to appear abruptly at the boundary between the corrosion product region and the quasi-brittle region. Thus, there should be a factor other than the direct contact of the crack tip with the corrosion solution, which drives the crack growth producing the quasi-brittle region. This factor should also be associated with the side surface corrosion product layer because the quasi-brittle region is absent on the fracture surfaces of the reference and corrosion product-free specimens. Furthermore, similarly to that responsible for the corrosion product region, this factor should be eliminated by storage in air, since the quasi-brittle region is found to be reduced under increasing time and temperature of air storage. Probably, the only factor which might act this way is hydrogen. 

As was mentioned above, the evolution of hydrogen is an inalienable part of the corrosion process of Mg in aqueous solutions. Hydrogen can be produced during pre-exposure of the specimen as well as during the subsequent air storage if the corrosion solution sealed within the corrosion product layer interacts with the bare metal of the specimen. Indeed, it was shown recently that a large portion of hydrogen gas evolved from the pre-exposed specimen during its 24 h storage in CCl_4_ [26]. The volume of this hydrogen summed with that of hydrogen extracted from the same specimen during subsequent gas analysis was twice as high as the volume of hydrogen extracted from the counterpart specimen subjected to the gas analysis right after pre-exposure. This was concluded to be evidence for the generation of hydrogen via the corrosion reaction, which occurs within the surface layer of the pre-exposed specimen stored in the inert environment. The part of hydrogen being produced both during pre-exposure and subsequent air storage can likely be accumulated inside the discontinuities of the side surface corrosion product layer or at the interface between this layer and the bare metal. Presumably, this hydrogen can stay within the corrosion product layer in the molecular and atomic forms. Along with time after extraction of the specimen from the corrosion solution, the weakly bonded part of this hydrogen desorbs from the corrosion product layer even at room temperature. This is witnessed by the low-temperature desorption peak in Figure 12b as well as by the naked-eye-visible hydrogen bubbles released from the specimen immersed in CCl_4_ right after pre-exposure [26]. The remaining part of hydrogen sitting within the corrosion product layer is bonded more strongly and, therefore, desorbs only at higher temperatures and longer times. At least a part of hydrogen extracted from the specimen below 300 °C during the thermal desorption analysis can be associated with this strongly bonded hydrogen. It is established that the concentration of hydrogen extracted below 300 °C correlates well with both ductility and fractographic features of the pre-exposed specimens; these features include the lengths of the PESCC zone, corrosion product region, and quasi-brittle region. The observed correlation favours the suggestion that hydrogen being stored inside the corrosion product layer and extracted at relatively low temperatures can be involved in the mechanism of PESCC. Apparently, the post-exposure air storage results in the desorption of hydrogen from the corrosion product layer, thus suppressing PESCC. The correlation between the concentration of hydrogen and the size of the corrosion product region indicates that there is also a relationship between the amount of hydrogen and the corrosion solution stored within the corrosion product layer. Probably, both substances are contained together inside the same collectors because the corrosion solution can generate hydrogen. 

As has been mentioned, it is unclear whether hydrogen is responsible for the intergranular crack growth producing the corrosion product region on the fracture surface or not. However, the formation of the quasi-brittle region is likely a hydrogen-assisted process. Various morphological signatures of ductile fracture, which are typical of the quasi-brittle region, indicate that plastic deformation should be involved to some extent into the mechanism of hydrogen-assisted cracking observed. Taking into account that adsorbed hydrogen, rather than absorbed one, plays the key role in this process, one can plausibly suggest that AIDE is the most probable mechanism of the crack growth producing the quasi-brittle region. This mechanism implies that the dislocation emission from the crack tip is facilitated due to the adsorption of hydrogen atoms, thus promoting the locally ductile crack growth [21,41]. The propagation of such crack produces fine slip markings, dimples, and tear ridges on the fracture surface, which represent the time-extended plastic processes rather than instant brittle failures. It was also suggested that AIDE is responsible for the fluted facets on the fracture surface of pure Mg failing due to SCC [39]. A similar fluted morphology is also commonly observed within the quasi-brittle region on the fracture surface of the ZK60 and AZ31 alloys SSRT tested in the corrosion solution [24,42] or after pre-exposure [6,20,23].

### 3.7. Microstructural Transformations

As has been shown above, the long post-exposure storage in air at temperatures above 100 °C affects the strength of the alloy ZK60 in the reference and corrosion product-free state. This degradation of strength is likely attributed to some ageing-related microstructural transformations, which are not completed during the preceding short vacuum annealing at 250 °C. It is important to note that there is an apparent interplay between these transformations in the microstructure and the PESCC phenomenon occurring in the alloy. This can be clearly traced by the anomalous behaviour of mechanical properties, fractographic features, and hydrogen concentration at around 120 °C on the graphs shown in Figure 2, Figure 4a, Figure 9a and Figure 12a. Among all fractographic characteristics, the number of cracks is affected by these microstructural transformations to the greatest extent. Although the exact nature of the microstructural transformations and their role in PESCC were not investigated in the present study, a probable explanation for the observed results can be proposed. It is known that the mechanical properties of the alloy ZK60 can be influenced by ageing at temperatures above 100 °C [43,44] due to precipitation of some secondary phase particles such as Mg_2_Zn_3_ [45]. It has been reported that the precipitation of such particles leads to the increase in strength and hardness of Mg alloys in general and in ZK60 in particular, provided that the specimens are not overaged [46]. Otherwise, the coalescence of secondary phase particles results in the concomitant decrease in strength [43,44]. In particular, it was shown that ageing at 150 °C induced the reduction in strength and hardness of ZK60 if ageing lasted longer than 20 h [46]. Thus, the observed decrease in strength of ZK60 after 24 h of air storage above 100 °C is quite reasonable. Since most of the secondary phase particles in Mg alloys are noble with respect to the Mg matrix, they commonly provoke local anodic dissolution of surrounding metal submersed to an electrolyte. The corrosion pits produced at such anodic sites act as favourable nucleation points for the stress corrosion cracks [47]. Thus, assuming that the corrosion solution is likely to present within the layer of corrosion products and considering that it interacts with the bare metal during SSRT testing, it is reasonable to expect the greater number of cracks in the pre-exposed specimens to have a larger volume fraction of secondary phase particles in the microstructure. The greater number of active corrosion sites should also produce more hydrogen gas and corrosion products; this is supported by the anomalous increase in the concentration of hydrogen extracted in the temperature intervals of 24–300 and 300–450 °C, respectively.

### 3.8. The Scope of Further Research

Experimental results of the present study as well as the proposed discussion on the mechanisms of PESCC are specifically relevant to the ZK60 alloy. Since this alloy is representative of a broad class of wrought Mg alloys, one can likely expect the similar PESCC and SCC behaviour in other alloys of this class of materials. Nevertheless, the additional data pertinent to these phenomena occurring in Mg alloys of other chemical compositions is largely demanded to gain deeper understanding the generality of the observed phenomena on the one hand and to relate them to the chemistry of the alloy interacting with the aggressive environment on the other. In particular, the effect of the side surface layer of corrosion products in the mechanism of PESCC in different Mg alloys is to be elucidated. Furthermore, some other materials, e.g., structural steels and Ti alloys, experience appreciable embrittlement after pre-exposure to aggressive environments [48,49,50,51]. This embrittlement is generally accepted to be due to diffusible hydrogen, which, however, plays a less important role in PESCC of Mg alloys, as was shown above. Thus, the generality of the findings gained in the present study in regard to the driven mechanisms of PESCC is to be clarified also on a more global scale accounting for a wider range of metallic materials. The more specific experimental aspects related to the PESCC phenomenon in Mg alloys, such as the nature of the individual peaks on hydrogen thermal desorption spectra, the relationship between the microstructure and PESCC, as well as the origin and the role of the corrosion solution contained within the corrosion product layer, are of particular interest and will be considered in the forthcoming studies. 

## 4. Conclusions

The results obtained in the present paper show that the pre-exposure stress corrosion cracking (PESCC) of the alloy ZK60 can be fully or partially inhibited by air storage after pre-exposure in corrosion solution. The increase in time and temperature of air storage promotes the suppression of PESCC. The complete elimination of PESCC of the alloy ZK60 pre-exposed to the 4% NaCl + 4% K_2_Cr_2_O_7_ corrosive solution for 1.5 h can be achieved by 24 h of storage in air at 150–200 °C. The suppression of PESCC due to air storage is accompanied by the decrease in hydrogen concentration in the corrosion product layer and is manifested by the recovery of mechanical properties of the alloy as well as by the reduction in the brittle-like (PESCC) zone on the fracture surface, including both its peripheral region covered with corrosion products and the subsequent quasi-brittle region. It is suggested that the side surface layer of the corrosion product on the pre-exposed specimens serves as a “container” for the embrittling agents such as hydrogen and the corrosion solution, which interact with the bare metal during SSRT testing in air and are responsible for the formation of characteristic corrosion product region and quasi-brittle region on the fracture surface, respectively. The strong correlations between the length of the corrosion product region, the concentration of hydrogen extracted below 300 °C, and the extent of PESCC of the pre-exposed specimens suggest that both hydrogen and the corrosion solution stored within the side surface layer of corrosion products play vital roles in the mechanism of PESCC. The suppression of PESCC by air storage is due to the desorption of hydrogen and evaporation of the corrosion solution from the side surface layer of corrosion products, as is witnessed by the decrease in the hydrogen concentration as well as by the reduction in the size of characteristic regions on the fracture surface with the increasing temperature and time of the air storage. 

## Data Availability

The raw/processed data required to reproduce these findings cannot be shared at this time as the data also form part of an ongoing study. Specific requests can be directed to the corresponding author.
